# The Fibronectin Expression Determines the Distinct Progressions of Malignant Gliomas via Transforming Growth Factor-Beta Pathway

**DOI:** 10.3390/ijms22073782

**Published:** 2021-04-06

**Authors:** Chih-Wei Chen, Cheng-Han Yang, Yuan-Ho Lin, Ya-Chin Hou, Tain-Junn Cheng, Sheng-Tsung Chang, Yu-Hua Huang, Shang-Ting Chung, Chung-Ching Chio, Yan-Shen Shan, Hung-Chi Cheng, Wen-Tsan Chang

**Affiliations:** 1 Institute of Clinical Medicine, College of Medicine, National Cheng Kung University, Tainan 701, Taiwan; awei921@gmail.com (C.-W.C.); yachi2016@yahoo.com.tw (Y.-C.H.); ysshan@mail.ncku.edu.tw (Y.-S.S.); 2 Chi Mei Foundation Medical Center, Division of Neurosurgery, Department of Surgery, Tainan 710, Taiwan; chiocc@ms28.hinet.net; 3 Department of Occupational Safety and Health/Institute of Industrial Safety and Disaster Prevention, College of Sustainable Environment, Chia Nan University of Pharmacy and Science, Tainan 717, Taiwan; tjcheng@mail.chimei.org.tw; 4 Institute of Basic Medical Sciences, College of Medicine, National Cheng Kung University, Tainan 701, Taiwan; s58021014@mail.ncku.edu.tw (C.-H.Y.); rftest20336@yahoo.com (Y.-H.L.); 5 Chi Mei Foundation Medical Center, Department of Neurology and Occupational Medicine, Tainan 710, Taiwan; 6 Chi-Mei Foundation Medical Center, Departments of Pathology, Tainan 710, Taiwan; hoshifat@gmail.com (S.-T.C.); shiauyu428@gmail.com (Y.-H.H.); 7 Chi-Mei Foundation Medical Center, Departments of Nursing, Tainan 710, Taiwan; 8 Department of Biochemistry and Molecular Biology, College of Medicine, National Cheng Kung University, Tainan 701, Taiwan; 934308@gmail.com; 9 Division of General Surgery, Department of Surgery, College of Medicine, National Cheng Kung University, Tainan 701, Taiwan

**Keywords:** glioma, glioblastoma multiforme (GBM), tumor recurrence, tumor metastasis, epithelial-mesenchymal transition (EMT), fibronectin (FN), vimentin (VIM), transforming growth factor-beta (TGF-β)

## Abstract

Due to the increasing incidence of malignant gliomas, particularly glioblastoma multiforme (GBM), a simple and reliable GBM diagnosis is needed to screen early the death-threaten patients. This study aimed to identify a protein that can be used to discriminate GBM from low-grade astrocytoma and elucidate further that it has a functional role during malignant glioma progressions. To identify proteins that display low or no expression in low-grade astrocytoma but elevated levels in GBM, glycoprotein fibronectin (FN) was particularly examined according to the mining of the Human Protein Atlas. Web-based open megadata minings revealed that FN was mainly mutated in the cBio Cancer Genomic Portal but dominantly overexpressed in the ONCOMINE (a cancer microarray database and integrated data-mining platform) in distinct tumor types. Furthermore, numerous different cancer patients with high FN indeed exhibited a poor prognosis in the PrognoScan mining, indicating that FN involves in tumor malignancy. To investigate further the significance of FN expression in glioma progression, tumor specimens from five malignant gliomas with recurrences that received at least two surgeries were enrolled and examined. The immunohistochemical staining showed that FN expression indeed determined the distinct progressions of malignant gliomas. Furthermore, the expression of vimentin (VIM), a mesenchymal protein that is strongly expressed in malignant cancers, was similar to the FN pattern. Moreover, the level of epithelial–mesenchymal transition (EMT) inducer transforming growth factor-beta (TGF-β) was almost recapitulated with the FN expression. Together, this study identifies a protein FN that can be used to diagnose GBM from low-grade astrocytoma; moreover, its expression functionally determines the malignant glioma progressions via TGF-β-induced EMT pathway.

## 1. Introduction

Glioblastoma multiforme (GBM) is a primary neuroepithelial cancer in the central nervous system (CNS). This cancer accounts for 12% to 15% of all intracranial tumors and about 50% of astrocytomas [1]. Patients with GBM have a poor prognosis of just 12–15 months following standard therapy, with only 3–5% of patients surviving up to five years after diagnosis [2,3]. The current GBM treatment includes maximal resection, followed by radiotherapy with concomitant and adjuvant Temozolomide (TMZ) chemotherapy [4]. Despite these aggressive therapeutic regimens, GBM’s poor prognosis is mainly due to its high propensity for tumor recurrence. Its recurrence is inevitable after a median survival time of 32 to 36 weeks [5,6]. The recurrence most often occurs in the form of a local continuous growth within 2 to 3 cm from the border of the original lesion [7,8,9]. Choucair et al. reported that more than 90% of patients with glioma show recurrence at the original tumor location and the multiple lesions develop in 5% after standard treatment [6]. In spite of this, the extracranial GBM metastases are rare, only 0.4 to 0.5% [10]. Thus, the prognostic biomarkers about cancer outcomes (e.g., disease recurrence and progression, and overall survival) are important for the treatment and management of GBM patients.

The extracellular matrix (ECM) plays an important role in numerous cellular functions during normal and pathological processes, such as differentiation, apoptosis, neurite outgrowth, tumor invasion, and metastasis [11,12,13]. Glioma cells seem to create a permissive environment for invasion through attachment to the ECM via cell surface receptors and subsequent ECM degradation [14]. In addition, glioma cells synthesize and deposit ECM proteins, such as tenascin-C (TN-C), laminin (LM), fibronectin (FN), and type IV collagen (C-IV), which facilitate the tumor cell motility [14]. FN is a glycoprotein found in ECM as aggregates or fibrils [13,15,16]. It has many biological functions including cell adhesion, migration, and invasion, and mediates a variety of adhesive events by binding to fibrinogen/fibrin, collagen, heparin sulfate, and hyaluronic acid. FN is found at the gliomesenchymal junction of tumors, in tumor-associated blood vessels [17], and focally within and around glioma cells in situ [18], and is expressed by GBM cell lines in vitro [19]. The glioma cells express FN receptors, cluster around FN in vivo, and migrate in response to FN in vitro [20]. In addition, of several ECMs tested, GBM cells migrate most efficiently on FN [21]. Migration along FN positive mesenchymal cells may lead to the gathering of glioma cells in the perivascular regions and along the meninges [22].

Intermediate filament proteins are the main cytoskeletal proteins that play distinct roles under a variety of conditions. Vimentin (VIM), a 57-kDa protein, is belonged to type III of six different intermediate filament families [23]. It is normally present in cells of mesenchymal origin from the nucleus to the plasma membrane [24], and it provides cell structural support and maintains tissue integrity [25]. Moreover, it is also expressed in epithelial cells and is associated with metastatic disease [26,27], endothelial cell adhesion [28], migration, and/or invasive properties [27,29]. The VIM expression is detected in cancer cell lines and in most tumor types and is significantly associated with a poorer differentiation grade in lung cancers [30,31,32]. In addition, it has been reported the close relationship between the VIM expression level and local recurrence in oral squamous cell carcinomas [33,34]. In particular, VIM is also regarded as a marker of epithelial–mesenchymal transition (EMT), and the upregulation of VIM expression is observed in tumor types, such as prostate and breast cancers, malignant melanoma, and CNS tumors [35].

EMT is a process in which epithelial cells lose apical–basal polarity and cell-to-cell contacts but gain a mesenchymal phenotype, including increased cell-to-ECM contacts and cell migration [13,36,37]. This process decreases the expression of epithelial markers such as E-cadherin and increases the expression of mesenchymal proteins such as FN, N-cadherin, VIM, and the matrix metalloprotease (MMP)-2. FN is a marker for EMT that occurs during development and has been linked to tumor malignancy. During EMT, epithelial cell adhesion switches from cell–cell contacts to mainly cell–ECM interactions raising the possibility that FN may have a role in promoting this transition. EMT can be induced or regulated by various growth and differentiation factors such as transforming growth factor-beta (TGF-β), growth factors that act via receptor tyrosine kinases, including fibroblast growth factor, hepatic growth factor, and platelet-derived growth factor, and Wnt and Notch proteins [38]. Among these, TGF-β has received more attention as a key inducer of EMT during embryogenesis, tissue fibrosis, and cancer progression. The induction of EMT by TGF-β is first observed in cell culture. Upon TGF-β treatment, epithelial cells changed from cuboidal to an elongated spindle shape and showed decreased expression of epithelial markers and enhanced expression of mesenchymal proteins such as FN and VIM [39]. This evidence implicates increased TGF-β signaling as a major effector of EMT in tumor progression and metastasis [40]. Cancer cells often increase their production of active TGF-β, which triggers EMT, allows the cells to become motile and invasive, and enhances angiogenesis in close proximity to the tumor microenvironment, providing an invasive route for cancer cell metastasis [13,41].

Iwadate et al. reported that high expression of TGF-β around necrotic regions is significantly correlated with shorter progression-free survival and overall survival in patients with GBM [42]. In addition, Park and Schwarzbauer concluded that exogenous administration of FN induces an EMT response including upregulation of the EMT markers FN, Snail, N-cadherin, VIM, and MMP2, in addition to the acquisition of cell migratory behavior [43]. Moreover, they also suggested that FN enhances the effect of endogenous TGF-β to induce EMT and then increased levels of FN to facilitate tumorigenesis. While TGF-β is a well-known inducer of EMT, the contributions of the ECMs such as FN upregulation to this process are not well understood. Therefore, we hypothesized that the expression of EMT marker FN is activated during malignant glioma recurrence and progression. To examine this correlation, we first applied web-based open megadata minings, including the Human Protein Atlas, cBio Cancer Genomic Portal, ONCOMINE, and PrognoScan databases, to analyze directly the relationship between FN expression level and cancer patient outcome. Second, we retrograde reviewed clinical astrocytoma cases that received surgical resection with the pathologically proven to find out the types of progression including local recurrence, remote brain, and spinal metastases. Third, we used immunohistochemical (IHC) staining of FN, VIM, and TGF-β in the specimens isolated from the same patient received more than two times surgeries (origin and recurrence) to evaluate the correlation between FN expression and TGF-β-induced EMT in the malignant glioma patients with local recurrence, remote brain, or spinal metastasis.

## 2. Results

### 2.1. Identification of Potential Protein Biomarker for GBM

**Figure 1:**
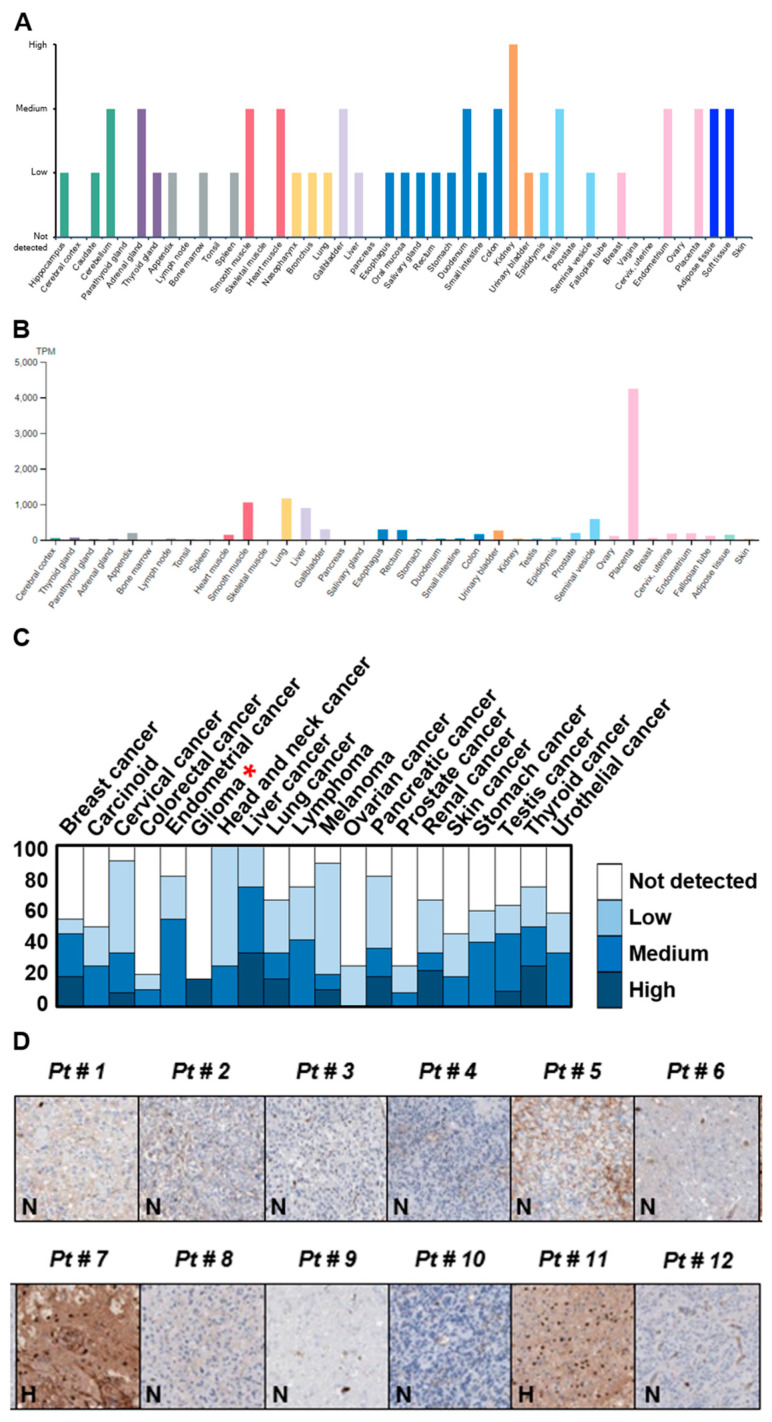
Fibronectin (FN) displays low-to-medium levels in distinct tissues and organs but exhibits either no or high expression in gliomas. (**A**) Protein and (**B**) RNA levels of FN in different tissues and organs obtained from the Human Protein Atlas database (https://www.proteinatlas.org/, accessed on 1 November 2017) mining. (**C**) Statistical pattern of FN expression in distinct cancer types by immunohistochemical (IHC) staining acquired from the Human Protein Atlas database mining. (**D**) Expression of FN in 12 glioma specimens by IHC staining provided by the Human Protein Atlas database mining. Red * indicates the statistical expression pattern of FN in glioma tumors.

**Figure 2:**
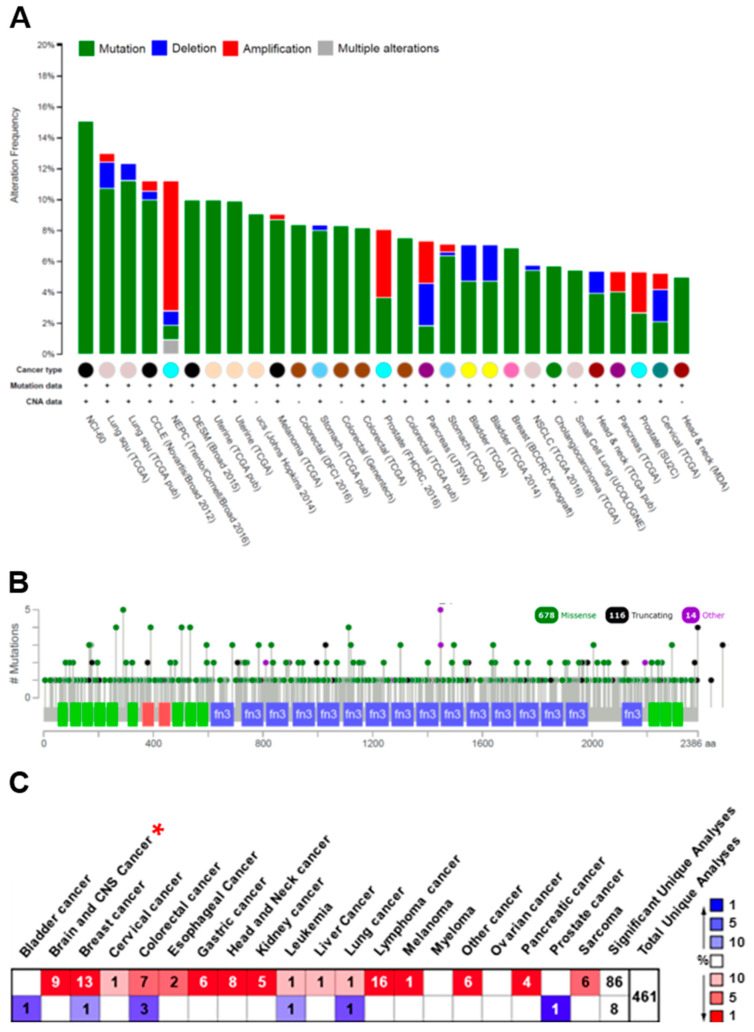
FN is mainly mutated in the genomic DNA but dominantly overexpressed in the gene expression in many different cancer types. (**A**) The type and (**B**) distribution of FN genomic DNA alteration in numerous distinct tumor types analyzed from the cBio Cancer Genomic Portal database (http://www.cbioportal.org/, accessed on 1 November 2017) mining. (**C**) The statistical FN expression levels compared between normal and cancerous tissues analyzed from the ONCOMINE database (https://www.oncomine.org/resource/, accessed on 1 November 2017) mining in different cancer types. Red * indicates the statistical expression pattern of FN in glioma tumors.

**Figure 3:**
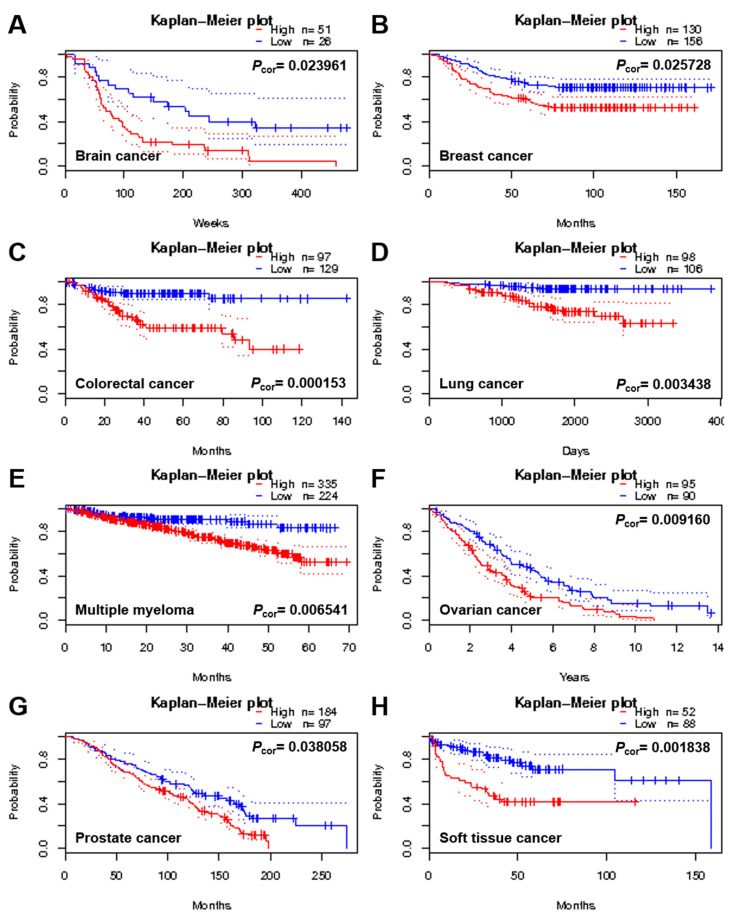
A high level of FN expression is frequently correlated with poor prognosis in numerous distinct tumor types. Survival analysis of FN expression level in different cancer types, including brain (**A**), breast (**B**), colorectal (**C**), lung (**D**), multiple myeloma (**E**), ovarian (**F**), prostate (**G**), and soft tissue (**H**) cancers, by the PrognoScan database (http://www.abren.net/PrognoScan/, accessed on 1 November 2017) mining. The red dotted line indicates high level of FN expression and the blue dotted line indicates low level of FN expression. The difference between the two groups of low and high expression levels was recognized as statistically significant when *p*-value was <0.05.

The prognostic biomarkers about the outcome (e.g., disease recurrence, progression, and overall survival) of cancers particularly malignant tumors such as GBM are important for the design of strategy on patient’s treatment and management. In an attempt to identify proteins that display low or no expression in low-grade astrocytomas but a high level in GBM, a cancer-related and multifunctional glycoprotein FN was specially chosen and examined according to the mining of the Human Protein Atlas database (https://www.proteinatlas.org/, accessed on 1 November 2017). The results revealed that the FN expression in brain and CNS tissues was mainly low in both protein and RNA levels (Figure 1A,B). In addition, IHC staining of a total of 12 glioma specimens showed that only two malignant gliomas displayed a high level of FN expression, and the other 10 gliomas were not detected in FN expression (Figure 1C,D). To evaluate fully whether the expression of the FN is linked with tumor progression and/or malignancy, the genomic structure and expression level of FN were examined and analyzed using web-based open megadata minings. The cBio Cancer Genomic Portal database (http://www.cbioportal.org/, accessed on 1 November 2017) mining indicated that the FN genomic loci were mainly mutated in distinct tumor types with only very few amplifications and/or deletions (Figure 2A). In particular, the mutations were distributed and covered in the whole coding amino acid sequence without any specific pattern or hotspot; in addition, the mutation types were major in missense mutations and minor in truncating mutations (Figure 2B). However, the ONCOMINE database (https://www.oncomine.org/resource/, accessed on 1 November 2017) analysis revealed that the FN gene was dominantly overexpressed in many different cancer types, including brain and CNS, breast, gastric, head and neck, kidney, lymphoma, pancreatic, and other cancers (Figure 2C). In particular, the expression level of FN was ranked at the top 1% or 5% of the whole genome analysis in most tumor types. Moreover, the different cancer patients, including those with brain tumors with high FN expression, were associated with poor prognosis in the PrognoScan database (http://www.abren.net/PrognoScan/, accessed on 1 November 2017) (Figure 3) and the Human Protein Atlas database—Pathology Atlas (https://www.proteinatlas.org/, accessed on 1 November 2017) (data not shown) minings. These analyses strongly suggested that FN plays a role or directly involves in tumorigenesis and/or malignant progression of cancers.

### 2.2. Recruitment of Specific Brain Tumor Glioma Specimens

To analyze directly the relationship between FN expression and glioma progression and malignancy, the level of FN was examined and compared in the surgical specimens removed from the same patient with received more than two times surgeries (origin and recurrence). For this purpose, we retrospectively reviewed 151 malignant glioma cases with surgical, pathological diagnoses from 2009 to 2016 in the Chi Mei Foundation Medical Center (CMFMC). There were 91 GBMs (WHO grade IV), 36 anaplastic astrocytomas (WHO grade III), 6 grade II astrocytomas, and 18 grade I astrocytomas. The recurrent patterns of malignant gliomas are classified into three subtypes—local recurrence, remote brain metastasis (41), and spinal metastasis. Among the 151 malignant glioma cases, there were 144 (95%) local recurrences, 3 (2%) local recurrences with remote brain metastasis, 2 (1%) remote brain metastases, and 2 (1%) spinal metastases. Overall, 11 cases with recurrence that received at least two times surgeries were enrolled in the study. Among the 11 cases, there was one low-grade astrocytoma with GBM transformation, six GBMs with local recurrence, one GBM with local recurrence and then remote brain metastasis, two GBMs with spinal metastasis, and one GBM with local recurrence and then low-grade astrocytoma progression. The tumor specimens removed from five patients with surgically treated, histologically proven astrocytoma or GBM, including a low-grade astrocytoma with local GBM transformation, a GBM with local recurrence, a GBM with local recurrence and then remote brain metastasis, a GBM with spinal metastasis, and a GBM with local recurrence and then low-grade astrocytoma progression, were examined by IHC staining with an antibody specifically against FN. In addition, the IHC staining of both VIM and TGF-β was also performed to identify the TGF-β-induced EMT pathway. The tumor specimens from just five patients were examined by IHC staining with an antibody specifically against FN, VIM, and TGF-β. Due to the incomplete collection of Institutional Review Board (IRB) informed consent, not all of the 11 cases were examined in this study. However, this study did particularly include distinct specimens in each malignant glioma recurrent pattern (Table 1). The results obtained from this experiment were described in the following sections according to the progression of malignant gliomas.

### 2.3. A Low-Grade Astrocytoma with Local GBM Transformation

**Figure 4:**
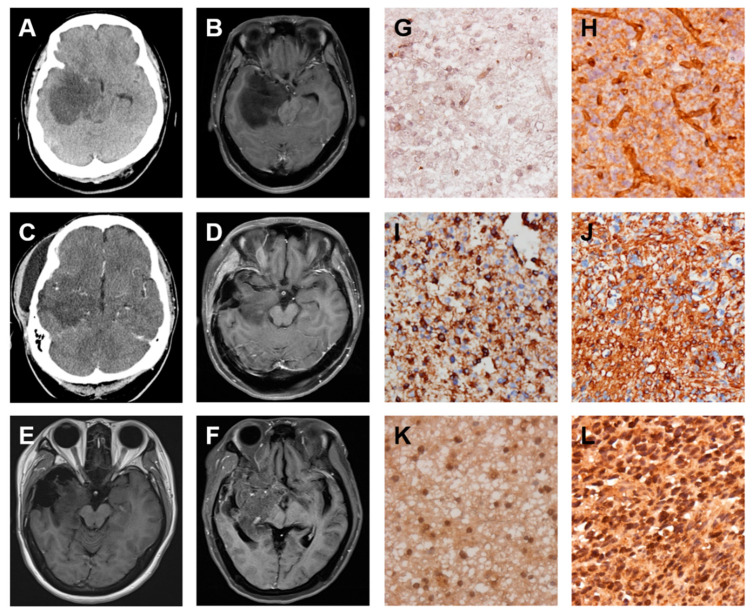
FN, vimentin (VIM), and transforming growth factor-beta (TGF-β) expressions are increased during the progression of low-grade astrocytoma into glioblastoma multiforme (GBM). Serial images including first and follow-up examinations of a patient with GBM are shown by using either brain computerized tomography (CT) on 25 October 2010 (**A**) and 8 December 2010 (**C**) or head MRI on 25 October 2010 (**B**), 5 May 2011 (**D**), 28 December 2012 (**E**) and 17 May 2013 (**F**). The IHC staining images indicate the expression level of FN (**G** and **H**), VIM (**I** and **J**), and TGF-β (**K** and **L**) proteins in the origin of low-grade astrocytoma (**G**, **I**, and **K**) and local recurrence of GBM (**H**, **J**, and **L**) tumor specimens.

A female 40 years of age was admitted to the CMFMC on 25 October 2010 due to a headache for about three months. Neurologic examination revealed no focal neurological deficit. Her past history showed no systemic disease and no vascular risk factors such as hypertension, hyperlipidemia, smoking, and diabetes. The results of laboratory studies were normal. Brain computerized tomography (CT) (Figure 4A) and magnetic resonance imaging (MRI) (Figure 4B) showed a right temporal mass lesion with significant mass effect. Craniotomy with tumor removal was performed on 26 October 2010. Its pathology was low-grade astrocytoma. Repeated follow-up CT on 8 December 2010 (Figure 4C) revealed a residual tumor. Therefore, the patient received a full course of radiotherapy (RT): 5400 cGy from 13 December 2010 to 21 January 2011. Repeated follow-up MRI on 5 May 2011 (Figure 4D) showed the tumor shrinkage. Repeated follow-up MRIs on 25 November 2011 (data not shown), 1 June 2012 (data not shown), and 28 December 2012 (Figure 4E) revealed the tumor in stationary status. The patient was brought to the hospital because of general weakness and left side weakness on 17 May 2013. The brain MRI showed tumor recurrence with mass effect (Figure 4F). Craniotomy with tumor removal was carried out on 21 May 2013. Its pathology was GBM. The patient received Temodal treatment since 7 June 2013. Finally, the patient died on 28 October 2013. The original and recurrent tumor specimens obtained from surgical dissections were analyzed by IHC staining of FN protein. The results revealed that the original low-grade astrocytoma specimen displayed weak staining of FN protein (Figure 4G) but the recurrent GBM specimen exhibited moderately strong staining of FN protein (Figure 4H), indicating that the FN expression level is increased during malignant transformation of brain cancer. In addition, this malignant progression was further confirmed by IHC staining of VIM, a mesenchymal protein that is strongly expressed in malignant cancers. The expression level of VIM was similar to the expression pattern of FN (Figure 4I,J). Moreover, this malignant progression was correlated directly with the upregulation of the EMT inducer TGF-β expression (Figure 4K,L).

### 2.4. A GBM with Local Recurrence

**Figure 5:**
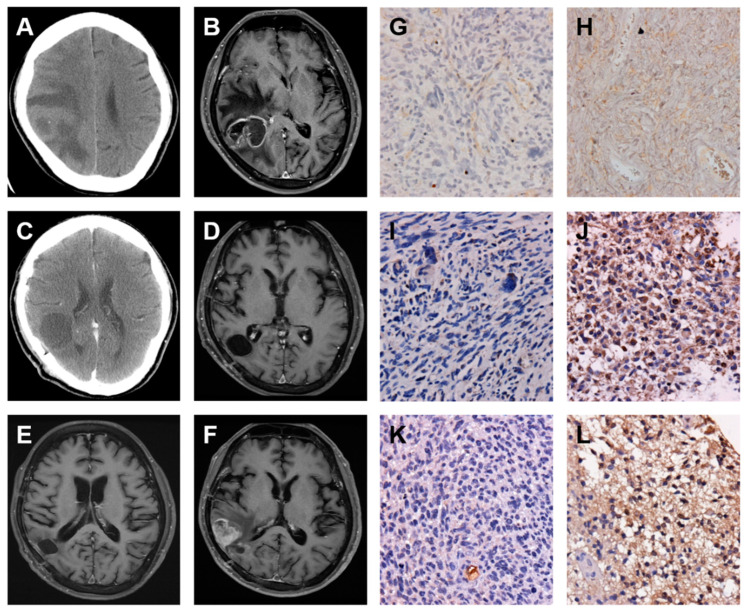
Enhanced FN, VIM, and TGF-β expressions are associated with the local recurrence of GBM. Serial images including first and follow up examinations of a patient with GBM are shown by using either brain CT on 10 July 2009 (**A**) and 7 August 2009 (**C**) or head MRI on 10 July 2009 (**B**), 30 November 2009 (**D**), 16 April 2010 (**E**), and 26 November 2010 (**F**). The IHC staining images indicate the expression level of FN (**G** and **H**), VIM (**I** and **J**), and TGF-β (**K** and **L**) proteins in origin (**G**, **I**, and **K**) and local recurrence (**H**, **J**, and **L**) of GBM tumor specimens.

A man 52 years of age was admitted to the CMFMC on 10 July 2009 because of headache and seizure for about one month. Neurologic examination revealed no focal neurological deficit. His past history showed no systemic disease and no vascular risk factors such as hypertension, hyperlipidemia, smoking, and diabetes. The results of laboratory studies were normal. Brain CT (Figure 5A) and MRI (Figure 5B) revealed a right parietal mass lesion with a significant mass effect. Craniotomy with tumor removal was performed on 14 July 2009. Its pathology was GBM. The patient received a full course concomitant medication of chemoradiotherapy (CCRT): 6000 cGy from 17 August to 25 September 2009 and Temodal treatment since 17 August 2009. The follow-up CT on 7 August 2009 (Figure 5C) showed a right parietal cystic lesion, which could be a postsurgical change. Repeated follow-up MRIs on 30 November 2009 (Figure 5D) and 16 April 2010 (Figure 5E) revealed the tumor in stationary status. The patient was brought to the hospital due to progressive headache and seizure on 26 November 2010. The brain MRI showed GBM local recurrence (Figure 5F). Craniotomy with tumor removal was carried out on 7 December 2010. Its pathology was recurrent GBM. The patient received continuous Temodal treatment since the first CCRT therapy. Palliative treatment was given. Ultimately, the patient died on 8 March 2011. The original and recurrent tumor specimens acquired from surgical dissections were examined by IHC staining of FN protein. The results showed that the original GBM specimen displayed a moderate staining of FN protein (Figure 5G), and the recurrent GBM specimen exhibited moderately strong staining of FN protein (Figure 5H), suggesting that the FN expression level is enhanced in the local recurrence of GBM. Furthermore, this local GBM recurrence was closely correlated with the EMT pathway through upregulation of both VIM (Figure 5I,J) and TGF-β (Figure 5K,L) expressions.

### 2.5. A GBM with Local Recurrence and Then Remote Brain Metastasis

**Figure 6:**
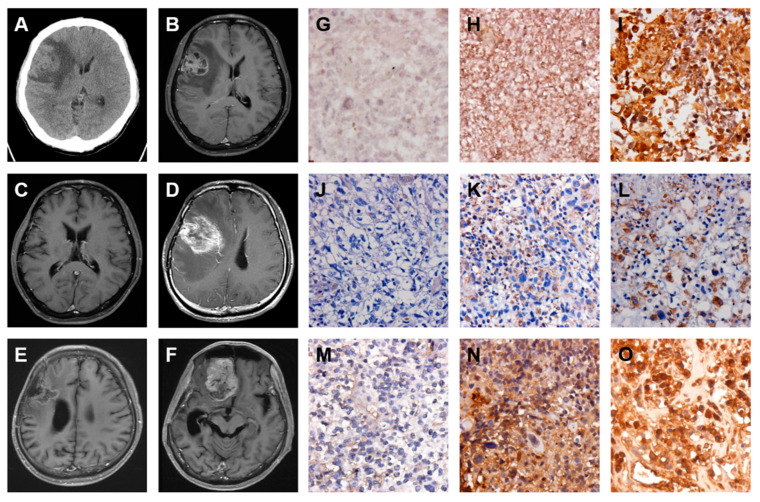
Expression of FN, VIM, and TGF-β is elevated during local recurrence and then remote brain metastasis of GBM. Serial images including first and follow up examinations of a patient with GBM are shown by using either brain CT on 1 August 2010 (**A**) or head MRI on 1 August 2010 (**B**), 11 April 2011 (**C**), 19 July 2012 (**D**), 30 January 2013 (**E**) and 3 June 2013 (**F**). The IHC staining images indicate the expression level of FN (**G**–**I**), VIM (**J**–**L**), and TGF-β (**M**–**O**) proteins in origin (**G**, **J**, and **M**), local recurrence (**H**, **K**, and **N**) and then remote brain metastasis (**I**, **L**, and **O**) of GBM tumor specimens.

A man 44 years of age was admitted to the CMFMC on 1 August 2010 due to headache and vomiting for about one month. Neurologic examination revealed no focal neurological deficit. His past history showed no systemic disease and no vascular risk factors such as hypertension, hyperlipidemia, smoking, and diabetes. The results of laboratory studies were normal. Brain CT (Figure 6A) and MRI (Figure 6B) showed a right frontal mass lesion with a significant mass effect. Craniotomy with tumor removal was performed on 3 August 2010. Its pathology was GBM. The patient received a full course concomitant medication of CCRT: 6000 cGy from August 30 to 8 October 2010 and Temodal treatment since 30 August 2010. Repeated follow-up MRIs on 11 April 2011 (Figure 6C) and 2 November 2011 (data not shown) revealed no recurrence of the GBM. The patient was brought to the hospital due to progressive headache and left side weakness on 19 July 2012. The brain MRI showed GBM local recurrence (Figure 6D). Craniotomy with tumor removal was carried out on 24 July 2012. Its pathology was recurrent GBM. The patient received a second-time full-course concomitant medication of CCRT: 6000 cGy from 10 September to 22 October 2012 with continuous Temodal treatment since the first CCRT therapy. Repeated follow-up MRI on 30 January 2013 (Figure 6E) revealed right frontal residual tumor but no obvious mass effect. Repeated follow-up MRI on 3 June 2013 (Figure 6F) showed regression of right frontal tumor but right frontal base tumor metastasis with mass effect. Craniotomy with right frontal base tumor removal was performed on 11 June 2013. Its pathology was metastatic GBM. The patient was kept under treatment with Temodal. Palliative treatment was given. Finally, the patient died on 8 January 2014. The original, recurrent, and remote brain metastatic tumor specimens received from surgical dissections were analyzed by IHC staining of FN protein. The results revealed that the original GBM specimen displayed moderate staining of FN protein (Figure 6G), the recurrent GBM specimen exhibited moderately strong staining of FN protein (Figure 6H) and the remote brain metastatic GBM specimen displayed very strong staining of FN protein (Figure 6I). These results indicated that the FN expression level was elevated during the progression of both local recurrence and remote brain metastasis. Moreover, this particular malignant progression was confirmed by IHC staining of both VIM and TGF-β expression levels. The results showed that the expression patterns of both VIM (Figure 6J–L) and TGF-β (Figure 6M–O) were very similar to the expression levels of FN during these distinct progressions of malignant gliomas.

### 2.6. A GBM with Spinal Metastasis

**Figure 7:**
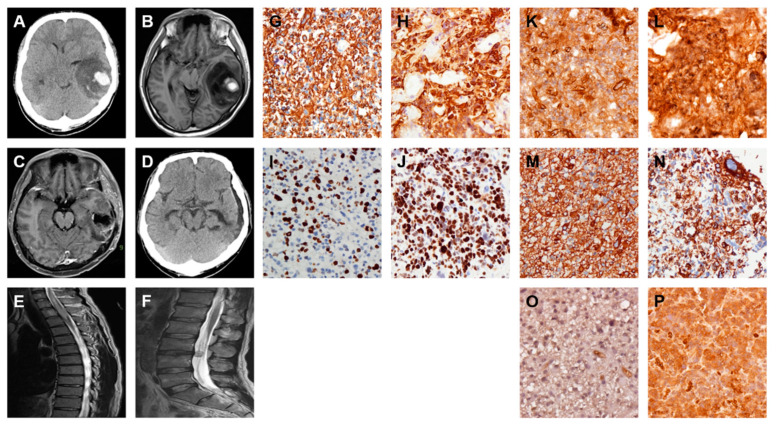
Increased FN, VIM, and TGF-β expressions are associated with spinal metastasis of GBM. Serial images including first and follow-up examinations of a patient with GBM are shown by using either brain CT on 11 May 2013 (**A**) and 17 August 2014 (**D**) or head MRI on 11 May 2013 (**B**), 26 July 2013 (**C**) and 17 August 2014 (**E** and **F**). The IHC staining images indicate the expression level of glial fibrillary acidic protein (GFAP) (**G** and **H**), Ki-67 (**I** and **J**), FN (**K** and **L**), VIM (**M** and **N**), and TGF-β (**O** and **P**) proteins in origin (**G**, **I**, **K**, **M**, and **O**) and spine metastasis (**H**, **J**, **L**, **N**, and **P**) of GBM tumor specimens.

A man 35 years of age was admitted to the CMFMC on 11 May 2013 because of headache and vomiting for one week. Neurologic examination revealed no focal neurological deficit. His past history showed no systemic disease and no vascular risk factors such as hypertension, hyperlipidemia, smoking, and diabetes. The results of laboratory studies were normal. Brain CT (Figure 7A) and MRI (Figure 7B) revealed a left temporal mass lesion with a significant mass effect. Craniotomy with tumor removal was performed on 13 May 2013. Its pathology was GBM. The patient received a full course concomitant medication of CCRT: 6000 cGy from 10 June to 23 July 2013 with Temodal treatment from 10 June 2013. Repeated follow-up MRI (Figure 7C) on 26 July 2013 showed no recurrence of the GBM. The patient was brought to the hospital due to progressive left side weakness on 17 August 2014. The emergent brain CT revealed no GBM recurrence (Figure 7D). The patient was admitted for further study. The spinal MRI revealed intramedullary lesion extending from T1 to T3 level and L3 to L4 level (Figure 7E,F). A T1 to T3 laminectomy was undertaken and followed by an extensive biopsy on 20 August 2014. The final pathology was consistent with GBM metastasis. Palliative treatment was given, and the patient died one month after the diagnosis of spinal metastasis.

This spinal metastasis from brain GBM was examined by IHC staining of glial fibrillary acidic protein (GFAP), an intermediate filament protein expressed in the CNS astrocyte cells. The results revealed that both the original GBM and spine metastatic specimens expressed GFAP strongly (Figure 7G,H). In addition, the malignancy of spinal metastasis was also evaluated using IHC staining of Ki-67 protein, a cell proliferation biomarker. The results showed that the spine metastatic specimen expressed Ki-67 protein strongly (Figure 7I,J). The original GBM and spine metastatic tumor specimens obtained from surgical dissections were analyzed by IHC staining of FN protein. The results revealed that the GBM displayed moderately strong staining of FN protein (Figure 7K), and the spinal metastasis exhibited very strong staining of FN protein (Figure 7L), indicating that the FN expression level is enhanced during the progression of spinal metastasis from brain GBM. In addition, this distal metastasis from the brain to the spine was strongly correlated with the EMT pathway via upregulation of both VIM (Figure 7M,N) and TGF-β (Figure 7O,P) expressions.

### 2.7. A GBM with Local Recurrence and Then Low-Grade Astrocytoma Progression

**Figure 8:**
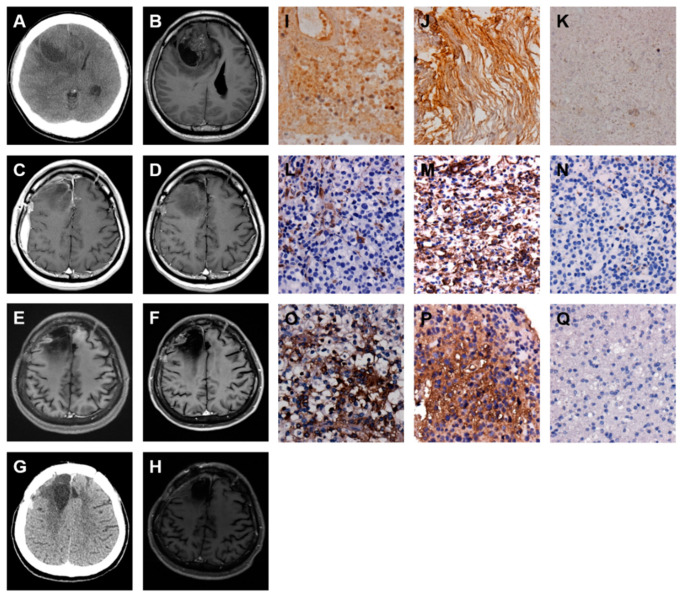
FN, VIM, and TGF-β expressions are increased in local recurrence but decreased during the progression of GBM into low-grade astrocytoma. Serial images including first and follow up examinations of a patient with GBM are shown by using either brain CT on 17 December 2009 (**A**) and 31 May 2016 (**G**) or head MRI on 17 December 2009 (**B**), 26 January 2010 (**C**), 4 June 2010 (**D**), 29 October 2014 (**E**), 30 October 2015 (**F**), and 13 September 2016 (**H**). The IHC staining images indicate the expression level of FN (**I**–**K**), VIM (**L**–**N**), and TGF-β (**O**–**Q**) proteins in origin (**I**, **L**, and **O**) and local recurrence (**J**, **M**, and **P**) of GBM, and progression into low-grade astrocytoma (**K**, **N**, and **Q**) tumor specimens.

A man 50 years of age was admitted to the CMFMC on 17 December 2009 due to diplopia and left side weakness for about two months. Neurologic examination revealed left side muscle power grade 4. His past history showed no systemic disease and no vascular risk factors such as hypertension, hyperlipidemia, smoking, and diabetes. The results of laboratory studies were normal. Brain CT (Figure 8A) and MRI (Figure 8B) revealed a right frontal mass lesion with a significant mass effect. Craniotomy with tumor removal was carried out on 22 December 2009. Its pathology was GBM. The follow-up MRI on 26 January 2010 (Figure 8C) showed a minimal residual tumor. Therefore, the patient received a full course concomitant medication of CCRT: 6000 cGy from 1 February to 16 March 2010 and Temodal treatment since 1 February 2010. The follow-up MRI on 4 June 2010 (Figure 8D) revealed the tumor shrinkage. Repeated follow-up MRIs on 17 September 2010 (data not shown), 8 March 2011 (data not shown), 15 November 2011 (data not shown), 18 October 2012 (data not shown), 6 December 2013 (data not shown), and 29 October 2014 (Figure 8E) showed the tumor in stationary status. The regular MRI follow-up on 30 October 2015 (Figure 8F) revealed a right frontal nodule lesion; a recurrent tumor was impressed. Craniotomy with tumor removal was performed on 6 January 2016. Its pathology was GBM. The patient kept Temodal treatment and received Avastin ( Bevacizumab) treatment since 16 February 2016. The brain CT follow-up on 31 May 2016 (Figure 8G) showed the tumor recurrence. Craniotomy with tumor removal was carried out on 1 June 2016. Its pathology was diffuse astrocytoma (WHO grade II). The follow-up MRI on 13 September 2016 (Figure 8H) revealed the tumor shrinkage. The patient’s general condition was well till now. The original GBM, recurrent GBM, and low-grade astrocytoma tumor specimens acquired from surgical dissections were analyzed by IHC staining of FN protein. The results showed that the original GBM displayed moderate staining of FN protein (Figure 8I), and the recurrent GBM exhibited moderately strong staining of FN protein (Figure 8J), but the recurrent low-grade astrocytoma displayed weak staining of FN protein (Figure 8K), indicating that the FN expression level is decreased strongly during the progression of low-grade astrocytoma from GBM. Interestingly, these very special expression patterns of FN in GBM local recurrence and then low-grade astrocytoma progression were almost recapitulated by the expression levels of both VIM (Figure 8L–N) and TGF-β (Figure 8O–Q). These results indicated that the change of FN expression during malignant glioma progressions was strongly correlated with the TGF-β-induced EMT pathway.

## 3. Discussion

GBM is the most common and malignant primary brain tumor in adults. The surgeons attempt to preserve neurological function and maintain patients’ quality of life; subtotal resections are sometimes carried out when tumors infiltrate eloquent areas of the brain. Tumor recurrence is also defined by the appearance of residual cancer cell growth on imaging studies or the manifestation of new clinical symptoms. The term “tumor recurrence” is frequently used synonymously with “cancer/tumor progression” because of the spectrum from which new lesions can develop. The recurrent brain tumors are classified into three subtypes—local recurrence, remote brain metastasis, and spinal metastasis [44]. More than 90% of patients with malignant glioma showed local recurrence, the remote brain metastasis developed in 5% of patients, and the spinal metastasis was less than 1% (10). In this study, among the 151 malignant glioma cases, there were 144 (95%) local recurrences, 3 (2%) local recurrences with remote brain metastasis, 2 (1%) remote brain metastases, and 2 (1%) spinal metastases. The prevalence of malignant gliomas recurrent patterns presented in this study is consistent with previous reports. Possible dissemination routes for remote brain metastasis of GBM are the following: meningeal–subarachnoid space, subependymal route, intraventricular route, and direct brain penetration [45]. The underlying pathophysiologic mechanism may be simultaneously neoplastic transformation [46,47].

Different molecular factors such as p53 mutation, Ki-67/MIB-1 labeling index, and O-6-methylguanine-DNA methyltransferase (MGMT) promoter methylation have been correlated with GBM recurrences [48,49]. Li et al. highlighted the differences in clinical features, molecular subtypes, and gene alterations between primary and recurrent GBMs [50]. The appearance of new genetic mutations or epigenetic aspects and malignant phenotypes in the process of recurrence increases the difficulty of treatment for recurrent GBM [51,52,53]. A glycoprotein FN is expressed by GBM cell lines in vitro and is also regarded as a marker of TGF-β-induced EMT [19,40]. Therefore, we hypothesized that the expression of FN is activated during GBM progression via the TGF-β-induced EMT pathway. Thus, IHC staining of FN, VIM, and TGF-β in paired human primary and recurrent malignant glioma specimens was used to evaluate the correlation between FN expression and the TGF-β-induced EMT pathway.

The intensity of expression for each IHC staining was assessed by using a semiquantitative four-grade system: Grade 0, no expression; Grade 1, minimal expression; Grade 2, moderate expression; Grade 3, marked expression with generalized or focal distribution [54]. The clinical information and the results of IHC staining were summarized in Table 2. This study showed that the FN expression is correlated with the distinct progressions of malignant gliomas. In addition, the expression of VIM, a mesenchymal protein that is strongly expressed in malignant cancers, was similar to the pattern of FN. Moreover, the level of EMT inducer TGF-β was recapitulated with the expression pattern of both FN and VIM. Together, this study identifies a protein biomarker FN that can be used to distinguish GBM from low-grade astrocytoma, and its expression has functional relevance to malignant glioma progressions via the TGF-β-induced EMT pathway. However, this study has a major limitation. The tumor specimens of recurrent and malignant gliomas enrolled and examined in this investigation are too small only five cases. To enhance the functional role of FN during malignant glioma progressions, the study should continually analyze the expression levels of FN, VIM, and TGF-β in recurrent and malignant glioma patients.

According to the canonical conception, TGF-β promotes EMT during cancer cell motility, invasion and metastasis; therefore, the EMT markers such as FN and VIM are induced. However, FN is a high-molecular-weight extracellular matrix glycoprotein that binds to membrane-spanning receptor proteins and therefore plays a major role in cell adhesion, growth, migration, and differentiation [55,56]. It elicits intracellular signaling by inducing integrin clustering that results in the recruitment and activation of tyrosine kinases including focal adhesion kinase (FAK), Src family kinases (SFK), and their substrates [57]. Therefore, FN plays an important role in the pathogenesis of cancer [13,58,59,60,61,62]. Ohnishi et al. demonstrated that FN is deposited in the extracellular matrix of tumors and plays a critical role in the biological behaviors of the cancer cells, particularly in FN-stimulated cell migration in vivo [63]. In recent studies, Park and Schwarzbauer explored that exogenous administration of FN induces an EMT response including upregulation of the EMT markers FN, Snail, N-cadherin, VIM, and MMP2, in addition to the acquisition of cell migratory behavior [43]. Sahoo et al. provided key insights into the role of ECM-derived TGF-β signaling to promote tumor metastasis [64]. Griggs et al. proposed a novel role for FN in EMT in which the assembly of FN serves to localize TGF-β1 signaling to drive EMT progression [65]. Although this study cannot reveal the causal relationships between FN and TGF-β, it does identify the FN expression that is consistent with the distinct progressions of malignant gliomas.

Kubelt et al. investigated the expression of Twist1, Snail1, Snail2/Slug, desmoplakin, biglycan, β-catenin, L1CAM, FN, VIM, and TGF-β1 with its receptors TGF-βR1 and TGF-βR2 in 17 matched probes of solid primary and recurrent human GBMs by real-time reverse transcription–polymerase chain reaction (RT-PCR) and by double-immunofluorescence staining to precisely identify EMT molecule-expressing cell types [66]. However, the expression of desmoplakin, VIM, FN, and TGF-β1 with its receptors TGF-βR1 and TGF-βR2 was almost unchanged. There may be sampling bias because the cellular architecture of GBMs is very heterogeneous, particularly when considering the aspect that the different cell types within GBMs, the tumor cells themselves, endothelial cells, and microglia/macrophages are all possible sources of EMT markers.

However, the majority of GBMs do not show intrinsic E-cadherin expression [67], the classical “E-cadherin to N-cadherin switch” is unlikely to correlate with EMT in GBMs [68], and therefore, the term glial-to-mesenchymal transition (GMT) is coined [69]. Mahabir et al. investigated the EMT process in pairs of primary and recurrent GBMs [69]. They found, by PCR analysis, that the expression of collagen, MMP-9, smooth muscle α-actin (α-SMA), CD44, FN, and YKL-40 are elevated in the recurrent glioma samples, and using IHC staining, they demonstrated that the expression level of VIM, α-SMA, and CD44 is increased in 22 cases of clinically recurrent gliomas [69]. However, they did not address the relevance of other processes involved in mesenchymal–epithelial transition (MET) or GMT.

## 4. Conclusions

Despite the advancement in therapeutic aspects, patients with GBM often exhibit a poor prognosis. In patients who have finished first-line treatment, strict tumor surveillance with regularly scheduled imagings and clinical evaluations may enable early detection of tumor recurrence and allow for immediate therapies. In contrast, this study is to immunohistochemically/semi-quantitatively assess the expression of FN in a retrospective series of recurrent malignant glioma specimens. As far as we know, this is the first investigation specifically addressed to immunohistochemically evaluate the expression of FN, VIM, and TGF-β in relation to the GBM progressions after standard treatments. The results indicated that TGF-β induces an EMT response with elevated expression of EMT markers and increased cell migratory behavior in patients with malignant glioma progressions. In addition, FN contributes to the development of EMT through cooperation with signaling initiated by the TGF-β. This study suggests that the malignant glioma cells expressed FN is elicited via a TGF-β pathway in correlation with distinct GBM progressions including local recurrence, remote brain, and spinal metastases. We propose that increased FN in malignant gliomas might be both a cause and a result of tumor initiation and/or progression. Therefore, modulating FN expression might present a new way to control GBM progressions.

## 5. Materials and Methods

### 5.1. Web-Based Open Megadata Minings

The biological nature of FN gene and protein in normal and cancerous human cells, tissues, and organs was analyzed by using the Human Protein Atlas database (https://www.proteinatlas.org/, accessed on 1 November 2017), mining with the keyword FN1 (fibronectin 1). The genomic alteration of the FN gene in cancerous human cells, tissues, and organs was examined by using the cBio Cancer Genomic Portal database (http://www.cbioportal.org/, accessed on 1 November 2017), mining with the keyword FN1. The statistical analysis of FN gene expression in cancerous vs. normal human tissues and organs was evaluated by using the ONCOMINE database (https://www.oncomine.org/resource/, accessed on 1 November 2017), mining with the keyword FN1. The prognostic value of FN expression level in cancerous human tissues and organs was analyzed by using the PrognoScan database (http://www.abren.net/PrognoScan/, accessed on 1 November 2017), mining with the keyword FN1.

### 5.2. Patients and Specimens

This study was assembled from patients who were histologically diagnosed with malignant gliomas and had undergone surgeries at the Department of Neurological Surgery, Chi Mei Foundation Medical Center (CMFMC), between 2009 and 2016. All medical records were reviewed retrospectively. The formalin-fixed, paraffin-embedded specimens from these patients were used for immunohistochemical (IHC) staining. The study was approved by the Ethics Committee of CMFMC. Written informed consent was obtained from the patients.

### 5.3. Immunohistochemistry (IHC) Staining

The expression level of GFAP, Ki-67, FN, VIM, and TGF-β proteins in brain tumor tissues was examined by IHC staining on paraffin sections. Tissues were deparaffinized and rehydrated followed by 120 °C for 40 min in retrieval buffer (pH9). Endogenous hydrogen peroxidase was blocked using 3% H_2_O_2_ for 30 min followed by nonspecific blocking with goat IgG (Jackson ImmunoResearch Laboratories Inc., West Grove, PA, USA) for 30 min. Sections were incubated with primary anti-GFAP, Ki-67, FN, VIM, and TGF-β antibodies (Sigma-Aldrich, Inc.; St. Louis, MO, USA) overnight at 4 °C. Next day, the tissues were incubated with a secondary antibody (Jackson) for 1 h at room temperature. The signal was detected by DAB chromogen solution (BioLegend, San Diego, CA, USA) and subsequent nuclear straining by hematoxylin (Merck, Darmstadt, Germany)). Images of sections were photographed by Zeiss Primo Star microscope (Carl Zeiss, Thornwood, NY, USA) at 40× or 400× magnifications.

## Figures and Tables

**Table 1:** Patient characteristics including age, sex, surgical date, and cancer location.

Type	Age/Sex	1st Surgery			2nd Surgery			3rd Surgery		
		Path	Date	Location	Path	Date	Location	Path	Date	Location
I	40/F	LGA	October 2010	R temporal	GBM	May 2013	R temporal			
II	52/M	GBM	July 2009	R parietal	GBM	December 2010	R parietal			
III	44/M	GBM	August 2010	R frontal	GBM	July 2012	R frontal	GBM	June 2013	R frontal
IV	35/M	GBM	May 2013	L temporal	GBM	August 2014	T spine			
V	50/M	GBM	December 2009	R frontal	GBM	January 2016	R frontal	LGA	June 2016	R frontal

I: A low-grade astrocytoma with local malignant GBM transformation; II: A malignant GBM with local recurrence; III: A malignant GBM with local recurrence and then adjacent brain metastasis; IV: A malignant GBM with spine metastasis; V: A malignant GBM with local recurrence and then low-grade astrocytoma progression. Path: Pathology; F: Female; M: Male; R: Right side; L: Left side; T: Thoracic; LGA: Low-grade astrocytoma; GBM: Glioblastoma multiforme.

**Table 2:** Patient characteristics and expression level of fibronectin, vimentin, and TGF-β.

Type	ST	1st Surgery				2nd Surgery				3rd Surgery			
		Path	FN *	VIM *	TGF-β	Path	FN *	VIM *	TGF-β	Path	FN *	VIM *	TGF-β
I	36	LGA	1	2	2	GBM	2	3	3				
II	20	GBM	1	1	1	GBM	2	2	2				
III	41	GBM	1	0	1	GBM	2	1	2	GBM	3	2	3
IV	15	GBM	2	2	1	GBM	3	2	3				
V	alive	GBM	2	1	2	GBM	3	3	3	LGA	0	0	0

ST: Survival time (months), from diagnosis to death.Path: Pathology; FN: Fibronectin; VIM: Vimentin; TGF-β: Transforming growth factor beta; LGA: Low-grade astrocytoma; GBM: Glioblastoma multiforme. * The expression intensity of IHC staining was assessed by using a semi-quantitative four-grade system: 0, no expression; 1, minimal expression; 2, moderate expression; 3, marked expression with generalized or focal distribution.

## Data Availability

Data available in a publicly accessible repository that does not issue DOIs.

## Abbreviations

α-SMA	smooth muscle α-actin
CCRT	concomitant medication of chemo-radio-therapy
C-IV	type IV collagen
CMFMC	Chi Mei Foundation Medical Center
CNS	central nervous system
CT	computerized tomography
ECM	extracellular matrix
EMT	epithelial–mesenchymal transition
FAK	focal adhesion kinase
FN	fibronectin
GBM	glioblastoma multiforme
GFAP	glial fibrillary acidic protein
GMT	glial to mesenchymal transition
IHC	immunohistochemical
LM	laminin
MGMT	O-6-methylguanine-DNA methyltransferase
MET	mesenchymal–epithelial transition
MMP	matrix metalloprotease
MRI	magnetic resonance imaging
RT	radio-therapy
RT-PCR	reverse transcription–polymerase chain reaction
SFK	Src family kinases
TGF-β	transforming growth factor-beta
TMZ	temozolamide
TN-C	tenascin-C
VIM	vimentin
